# Effect of Experimentally-Induced Trunk Muscular Tensions on the Sit-to-Stand Task Performance and Associated Postural Adjustments

**DOI:** 10.3389/fnhum.2017.00032

**Published:** 2017-02-06

**Authors:** Alain Hamaoui, Caroline Alamini-Rodrigues

**Affiliations:** ^1^Laboratoire de Physiologie de la Posture et du Mouvement, Centre universitaire de formation et de recherche Jean-François ChampollionAlbi, France; ^2^Laboratoire Mouvement, Equilibre, Performance, Santé (EA 4445), Université de Pau et des Pays de l’AdourTarbes, France

**Keywords:** muscular tension, mobility, trunk, sit-to-stand, focal movement, postural adjustments

## Abstract

It has been shown that increased muscular activity along the trunk is likely to impair body balance, but there is little knowledge about its consequences on more dynamic tasks. The purpose of this study was to determine the effect of unilateral and bilateral increases of muscular tension along the trunk on the sit-to-stand task (STS) performance and associated anticipatory postural adjustments (APAs). Twelve healthy females (23 ± 3 years, 163 ± 0.06 cm, 56 ± 9 kg), free of any neurological or musculoskeletal disorders, performed six trials of the STS at maximum speed, in seven experimental conditions varying the muscular tension along each side of the trunk, using a specific bimanual compressive load paradigm. A six-channel force plate was used to calculate the coordinates of the center of pressure (CP) along the anterior-posterior and medial-lateral axes, and the kinematics of the head, spine and pelvis, were estimated using three pairs of uni-axial accelerometers. The postural and focal components of the task were assessed using three biomechanical parameters calculated from CP signals: the duration and magnitude of APAs, and the duration of focal movement (dFM). Results showed that beyond a given level, higher muscular tension along the trunk results in longer APAs, but with a stable duration of the focal movement. In addition, no significant variation of APAs and FM parameters was found between bilateral and unilateral increases of muscular tension. It was suggested that restricted mobility due to higher muscular tension along the trunk requires an adaptation of the programming of APAs to keep the same level of performance in the STS task. These findings may have implications in treatment strategies aimed at preserving functional autonomy in pathologies including a rise of muscular tension.

## Introduction

According to the seminal work of Bouisset and Zattara (1981), voluntary movement induces a perturbation to balance, and a counter-perturbation must be developed for the movement to be performed efficiently. This counter-perturbation starts prior to the onset of the voluntary movement, and serves to create in the rest of the body a movement whose forces of inertia would, when the times comes, balance the inertia forces due to the voluntary movement. This early postural activity, termed anticipatory postural adjustments (APAs), is organized according to a reproducible pattern for a given movement and for every subject (Bouisset and Zattara, 1981, 1983). According to the hypotheses developed by Gelfand et al. (1966), the voluntary movement may be broken up into two components, one focal and one postural. The former refers to the voluntary movement itself, and the latter to the associated stabilizing activity of the body (Bouisset and Do, 2008). These two components require the mobility of a focal and a postural chain, which are usually located in distinct parts of the musculo-skeletal system. For example, the antepulsion of the upper limb is preceded by an acceleration of the shank (Bouisset and Zattara, 1983), and a bimanual isometric push task involves the mobility of the pelvis (Le Bozec and Bouisset, 2004). Consequently, restricted mobility of the bony chain may impair motor performance, even though its source is located in a part remote from the focal movement (FM).

According to Bouisset and Le Bozec (2002), postural chain dynamic mobility results from anatomical and physiological factors. The former ones refer to the structural properties of the bony chain (ligaments, bony stops…) which determine the articular range of motion, and can be considered as inherently passive. The latter and active factors are related to the pattern of muscular excitation, which can either generate or restrict bony segment displacements, depending on the agonist or antagonist function of the active muscles, respectively. In a pathological context, it has been assumed that a pain-related rise in muscular tension in low back pain is likely to impair the dynamic mobility capacity of the postural chain through a slowing-down effect (Hamaoui et al., 2004). Still for this pathology, it has also been shown an alteration of the APAs when performing upper limbs movements (Hodges and Richardson, 1999).

To date, a number of experiments have explored the effect of passive restrictions of postural chain mobility on voluntary movements. For example, a lower performance was shown to be associated with restricted pelvis mobility in a pointing task (Lino and Bouisset, 1994), in isometric pushing efforts (Le Bozec and Bouisset, 2004), or in the sit-to-stand task (STS; Diakhaté et al., 2013). However, few data are available on the influence of higher muscular tensions, although they represent a frequent symptom in a number of pathologies (hemiplegia, Parkinson’s disease, back pain, muscular spasms in sports medicine…). Based on different bimanual isometric pushing paradigms varying muscular activity along the trunk while seated (Hamaoui et al., 2007) and standing (Hamaoui et al., 2011; Hamaoui and Le Bozec, 2014), a number of studies support the view that muscular tension impairs body balance when it exceeds a given level, with a more significant effect in case of asymmetrical tensions. In line with these studies, the present work is aimed at exploring the effect of increased muscular tension along the trunk when performing a usual voluntary movement, the STS task. The STS task is considered as a very common daily activity (Burdett et al., 1985; Riley et al., 1991; Roebroeck et al., 1994), which is required for independent living (Inkster and Eng, 2004). It is defined as moving the mass center of the body upward, from a sitting to a standing position, without losing balance (Roebroeck et al., 1994). The STS involves a transition from an intrinsically stable three-point support to a dynamically stable two-point support and is considered in some respects as the most mechanically demanding functional task routinely undertaken during daily activities (Riley et al., 1991). Different strategies of kinematic and muscular patterns are possible (Doorenbosch et al., 1994; Rodrigues-de-Paula Goulart and Valls-Solé, 1999), with sagittal plane motion dominant (Shepherd and Gentile, 1994). Based on the above mentioned literature on postural chain mobility and muscular tension, it is hypothesized that higher muscular tensions along the trunk may cause the focal and postural components of the task to vary. This assumption will be explored using a biomechanical analysis of the STS task in different conditions varying muscular tension level and symmetry along the trunk.

## Materials and Methods

### Participants

Twelve healthy female subjects (age: 23 ± 3 years; weight: 56 ± 9 kg; height: 163 ± 0.05 cm, body mass index, BMI: 21 ± 3 kg/m^2^), free of any neurological or musculoskeletal disease took part in this study. To avoid any variation of muscular strength and spine mobility related to gender differences, we did not include male participants.

This study was carried out in accordance with the recommendations of the local “Ethics Committee for Human Movement Analysis”. All subjects gave written informed consent in accordance with the Declaration of Helsinki.

### Experimental Set-Up

#### Force Plate

A six-channel force plate (Bertec Corp., ref. 6012-15, Columbus, OH, USA), which collected the ground reaction forces and moments applied at its top surface was used to calculate the coordinates of the center of pressure (CP) along the anterior-posterior (Xp) and medial-lateral (Yp) axes, with the following formulas: Xp = My/Rz Yp = Mx/Rz (Rz is the vertical ground-reaction force; Mx and My are the moments around the anterior-posterior and medial-lateral axes, respectively).

**Figure 1:**
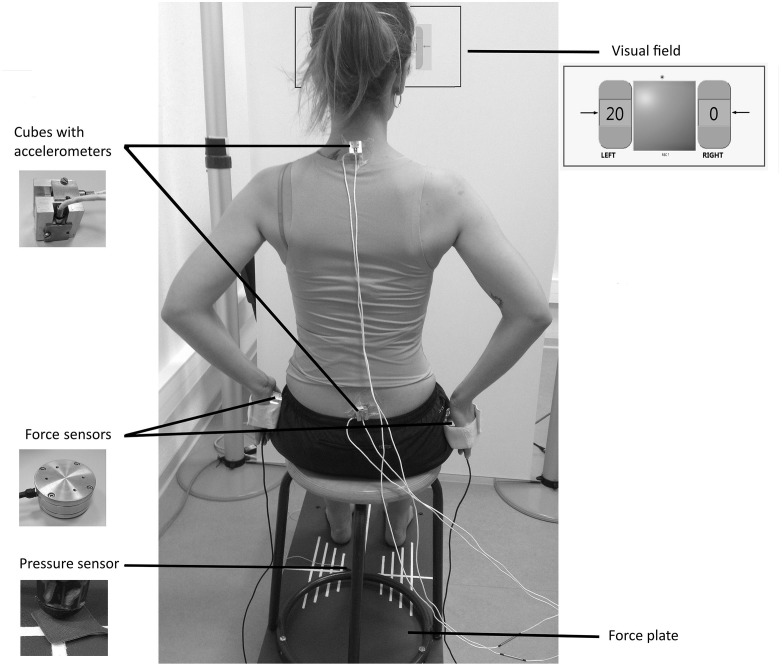
**Experimental set-up with the subject applying a unilateral compressive load at 20% of the maximum voluntary contraction (MVC)**.

A stool (height = 48 cm; depth = 39 cm) with four legs and a round wooden top (diameter = 30 cm) was screwed on to the force plate and used for the experiments (Figure 1). To keep constant the friction forces between the stool top and the surface contact of the body, all participants wore the same kind of shorts.

#### Pressure Sensor

A pressure sensor was placed under the left front leg of the stool to determine the “seat off” instant.

#### Accelerometers

Three pairs of mono-axial accelerometers (FGP sensors, ref XA1010-B, ±10 g, Les Clayes Sous Bois, France), were used to assess the local accelerations of the pelvis and trunk. Each pair was screwed on to a customized cube (length = 2 cm) with the two active axes located along the anterior-posterior and the vertical axes. The cubes were adhered to the skin with double-sided tape at the level of the first sacral vertebra and first thoracic vertebra (Figure 1).

#### Force Sensors

Two cylindrical load cells (FN 2114 model, FGP Sensors, Les Clayes Sous Bois, France) were used to set the active muscular tension at different levels alongside the torso when performing a bimanual compressive load paradigm (Figure 1). They were equipped with adjustable Velcro bands designed to keep each sensor in contact with the hand palm, and preventing it from falling.

#### Visual Field

The subject’s visual field consisted of a frontal white board, located 72 cm apart from the stool. A panel representing the tension generated on each load cell was screened on the board at the subject’s eye level, using a video-projector (Figure 1). The signals were displayed as a percentage of the maximum voluntary contraction (MVC) in a left and a right numerical indicator representing each sensor, and designed in Labview software (National Instruments). The background color of these indicators changed from red to green when the subject reached the required value, enriching the basic feedback by a specific indication of the task to perform. The experimentation room was lit with artificial lighting to obtain constant brightness.

#### Data Acquisition System

Data collected by the force plate, the pressure sensor, and the load cells were sampled at 200 Hz with a 16-bit A/D converter board (model CompactDAQ with 9215 modules, National Instruments, Austin, TX, USA), controlled by a custom code written in Labview software (National Instruments).

### Procedure

The participants sat on the platform with hips and knees approximately flexed to 90°, barefoot and feet apart. Adhesive tape was put around the feet outline in order to keep the same positioning for every trial. The load cells were positioned against hands palms and applied on the sides of the pelvis against the lateral surface of the trochanter major, with shoulders abducted to 35° (Figure 1). Subjects were asked to focus their gaze on the numerical indicators screened on the visual field and representing the load applied on the force sensors. This bimanual compressive load paradigm, which has been extensively described in a previous study (Hamaoui and Le Bozec, 2014) was considered as a reliable tool to control the active muscular tension along each side of the torso. Using an extensive surface EMG analysis, the authors found that the main muscles of this isometric task were latissimus dorsi, pectoralis major and thoracic erector spinae, while secondary muscles were identified as lumbar erector spinae, trapezius pars ascendez and obliquus externus abdominis. The mean EMG of the main muscles was reported to increase with the compressive load level, with a negligible activity toward the non-active side in unilateral loads.

Two 3-s trials in bilateral maximum compressive load were first performed to determine the MVC. Subjects had next to achieve the STS transfer while performing a compressive load in seven conditions varying the load level (0%, 20% and 40% of the MVC) and side (left, right, bilateral). It must be noted that for a compressive load at 0% (no force applied) the experimental instructions were the same for left, right and bilateral loads, and only one condition was considered. For each condition, the participants had to reach the requested compressive load parameters before starting the STS transfer and to keep them constant during the task, using the feedback screened on the visual field. This way, the variations of the muscular pattern that may be associated with the task completion were minimized.

The “STS” paradigm consisted in rising from the stool to reach the standing position as fast as possible, in response to a “Go” signal. A training period was used to familiarize the subjects with the paradigm before recording. Six 3-s runs were performed in every condition, with six additional runs in 0% for subsequent statistical analysis. The rest period was 30 s between runs and 1 min between series.

The order of the experimental condition was randomly assigned to avoid any order effect.

### Data Analysis

A careful visual inspection of CP curves along the anterior-posterior and medial-lateral axes was first performed for qualitative assessment (general time-course, curve shape). It was followed by a quantitative analysis of the APAs and FM of the STS. These two distinct phases were considered to be separated by the seat-off instant, for which the curve of the pressure sensor starts its fall towards zero. Three parameters were calculated from CP signals along the anterior- posterior axis:

– Duration of APAs (*dAPAs*): delay between the instant of seat-off and the first inflexion of the CP curve.
– Magnitude of APAs (Δ*Xp*): difference between the maximum and the minimum values of CP during the APAs phase.
– Duration of the focal movement (*dFM*): delay between the seat-off instant and the stabilization of the CP curve (beginning of the plateau region).

Data from the accelerometers were used to ensure that the head and trunk were kept still before the « Go » signal, and to discard trials in which the participants anticipated the instructions of the experimenter.

All parameters were calculated using a customized program written in MatLab software (The MathWorks, Inc., Natick, MA, USA).

Statistical analysis was performed using the Statistical Package for Social Sciences (SPSS) software V22 (Chicago, IL, USA). Normality of data distribution and the condition of sphericity were first checked using Shapiro-Wilk and Mauchly tests, respectively. A two-way repeated measures analysis of variance (ANOVA) was conducted for each dependent variable, with compressive load intensity (0%, 20% and 40% of the MVC) and laterality (bilateral, unilateral) as within-subjects factors. Values for the unilateral level were calculated from the mean between the left and right trials, as this study did not focus on specific left and right variations that might have resulted from handedness. When statistical significance was reached for compressive load intensity, the ANOVA was followed by a within-subjects analysis of contrasts (difference contrast) to compare the three levels of the independent variable. The level of statistical significance was set at *p* < 0.05.

## Results

### Qualitative Assessment of CP Curves

**Figure 2:**
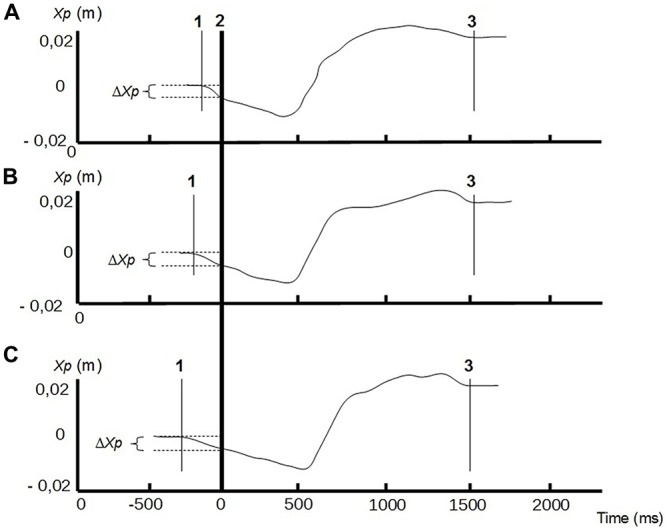
**Center of pressure (CP) traces along the anterior-posterior axis (Xp) of a representative subject, in different conditions of bilateral compressive load: no compression (A)** compressive load at 20% of the MVC **(B)**, compressive load at 40% of the MVC **(C)**. Line 1: CP onset; line 2: instant of « seat-off »; line 3: CP stabilization. Anticipatory postural adjustments (APAs) phase is between lines 1 and 2, and focal phase between lines 2 and 3. ΔXp represents the amplitude of the APAs.

The CP time course along the anterior-posterior axis revealed a typical shape which always starts with an almost linear backward displacement (beginning of the APAs), followed by a forward displacement with a steeper slope, and which ends with a less regular stabilization phase preceding the plateau (end of the FM; Figure 2). This pattern, which is consistent with data displayed in previous experiments (e.g., Schenkman et al., 1990; Diakhaté et al., 2013) was reproducible across subjects and conditions.

No such typical trace was found along the medial-lateral axis, with a high variability of CP displacements during APAs and FM. In addition, the scale of the signal was about ten times lower than along the anterior-posterior axis, and no specific lateral slope was found in relation to the side of the compressive load (left or right) in unilateral conditions.

### Quantitative Assessment of APAs and Focal Movement

**Figure 3:**
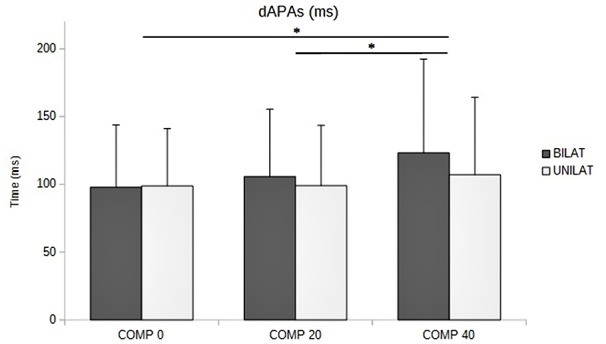
**Duration of APAs (dAPAs, in ms) as a function of the compressive load: means and standard deviations (error bars) are presented in unilateral (UNILAT) and bilateral (BILAT) compressive load, at 0% (COMP 0), 20% (COMP 20) and 40% (COMP 40) of the MVC**. *p < 0.05.

**Figure 4:**
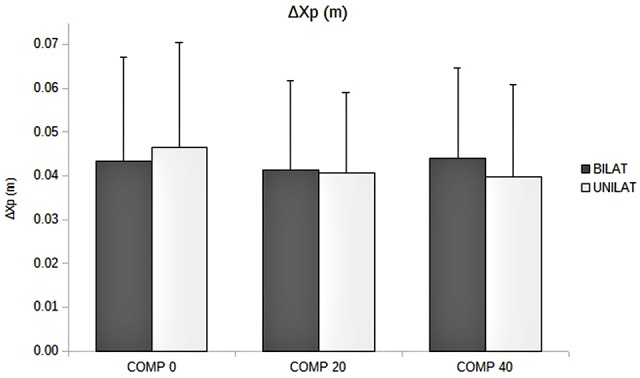
**Amplitude of APAs (ΔXp, in m) as a function of the compressive load: means and standard deviations (error bars) are presented in unilateral (UNILAT) and bilateral (BILAT) compressive loads, at 0% (COMP 0), 20% (COMP 20) and 40% (COMP 40) of the MVC**.

The ANOVA revealed that increased compressive load level was associated with a longer duration of the APAs, with a significant main effect (*p* < 0.05). Subsequent contrast analysis showed a significant variation between a compressive load at 40% (comp40) and at the two other levels, namely 0% (comp0) and 20% (comp20), but no substantial difference between comp0 and comp20 conditions (Table 1, Figure 3). However APAs magnitude (Δ*Xp*) and focal movement duration (dFM) were not sensitive to the level of the applied load (Table 1, Figure 4). These results were visible in CP traces, which exhibited longer APAs duration for comp40 and relative to comp20 and comp0, whereas APAs magnitude and FM duration remained stable (Figure 2).

**Table 1:** **Anticipatory postural adjustments (APAs) and focal movement (FM) parameters as a function of compressive load parameters**.

		COMP 0	COMP 20	COMP 40	*p(Comp main)*	*p(Comp 20/Comp 0*)	*p(Comp 40/previous*)	*p(Lat main)*
dAPAs (ms)	BILAT	98 ± 46	106 ± 50	123 ± 69	0.042	NS	0.048	NS
	UNILAT	99 ± 42	99 ± 44	107 ± 57
ΔXP (m)	BILAT	0.043 ± 0.024	0.041 ± 0.020	0.044 ± 0.020	NS	NS	NS	NS
	UNILAT	0.046 ± 0.024	0.041 ± 0.018	0.040 ± 0.021
dFM (ms)	BILAT	1693 ± 161	1641 ± 218	1593 ± 196	NS	NS	NS	NS
	UNILAT	1694 ± 190	1662 ± 222	1608 ± 150

*Mean ± SD of APAs duration (dAPAs, in ms), APAs magnitude (ΔXp, in m) and focal movement duration (dFM, in ms) are presented in bilateral (BILAT) and unilateral (UNILAT) compressive loads, at 0% (COMP 0), 20% (COMP 20) and 40% (COMP 40) of the maximum voluntary contraction (MVC). Results of statistical analysis display the main effect of the analysis of variance (ANOVA) for compressive load intensity (Comp main) and laterality (Lat main), and the contrast analysis for compressive load intensity (Comp 20 and Comp 40 relative to previous levels). NS: non significant*.

**Figure 5:**
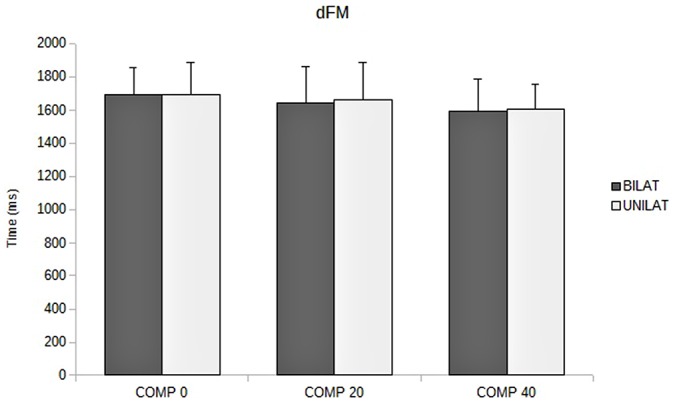
**Duration of focal movement (dFM, in ms) as a function of the compressive load: means and standard deviations (error bars) are presented in unilateral (UNILAT) and bilateral (BILAT) compressive loads, at 0% (COMP 0), 20% (COMP 20) and 40% (COMP 40) of the MVC**.

Compared to bilateral loads, unilateral loads resulted in smaller mean duration of APAs and longer mean duration of FM, but with no significant variation (Figures 3, 5, Table 1).

## Discussion

### Increased Muscular Tension Along the Torso Induces a Reorganization of the APAs

According to the analysis of dAPAs, higher muscular tension along the trunk resulted in longer APAs, with significant variations when the compressive load reached 40% of the MVC (comp40 relative to comp20 and comp0). This variation may be interpreted as a reorganization of the motor pattern, aimed at compensating for the lower capacity to generate counter-disturbing movements due to higher active muscular tension. It has already been assumed that postural chain muscular stiffening may slow down the counter-perturbing movements necessary to keep the body stable, and make them less efficient (Hamaoui et al., 2007). In a more conceptual point of view, it was also suggested that active muscular stiffening along the torso, when it exceeds a given level, is likely to alter the posturo-kinetic capacity (PKC; Hamaoui et al., 2011), which is defined as the ability to develop a counter-perturbation to the posture perturbation induced by segmental movement (Bouisset and Le Bozec, 1999). This reorganization of the APAs, which become longer but retains a similar magnitude, can be considered as efficient enough to keep constant the performance of the FM, as no variation was observed for FM duration. Longer APAs would allow more time to generate the inertia forces, which are necessary to balance the disturbing effect of the FM when the time comes, as hypothesized by Bouisset and Zattara (1981). With respect to the STS, it may favor the generation of the early propulsive impulse in the sagittal plane, which is an essential requirement of the task (Pai et al., 1994).

This variation of APAs duration has already been described in a previous study using the paradigm of shoulder flexion at maximum velocity, in different conditions varying the support base surface (Zattara and Bouisset, 1992). The authors showed that restricted support base surface induced longer APAs, which were in this case associated with a decrease of FM performance. Therefore, it can be hypothesized that the restriction of the PKC induced by increased muscular tension along the trunk in the present study, was relatively moderate and likely to be compensated for a reorganization of the APAs. This adaptability of the APAs has been described since the early works, with a variation of their duration and magnitude according to additional inertia loads (Bouisset and Zattara, 1981, 1983), support base configuration (Zattara and Bouisset, 1992; Lino and Bouisset, 1994; Yiou et al., 2007), and pelvic mobility (Le Bozec and Bouisset, 2004; Diakhaté et al., 2013).

### Unilateral and Bilateral Compressive Loads Did Not Impair the Focal Component of the STS

In contrast with previous studies describing lower body balance in case of increased trunk muscular tension in seated (Hamaoui et al., 2007) or standing (Hamaoui et al., 2011) postures, no variation of FM duration, and thus of performance, was observed in the present experiment. This difference might be attributed to the high levels of motor muscles activity (Rodrigues-de-Paula Goulart and Valls-Solé, 1999) and range of motion (Roebroeck et al., 1994) associated with the STS task, as compared to the low levels required for postural maintenance. Indeed, joint movements have been reported to be less than 1° while standing (Hodges et al., 2002), with a postural activity below 10% of the MVC (Okada, 1973). This low activity might be sensitive to postural muscles tensions, even limited by the maximum compressive load at 40% of the MVC. In contrast, the very dynamic displacements of the articulated chain required for the STS task, may overcome the slowing down effect induced by the compressive load paradigm.

Still in contrast with a study carried out during postural maintenance (Hamaoui and Le Bozec, 2014), the performance did not appear lower for unilateral loads, with similar FM duration between unilateral and bilateral conditions. Likewise, APAs duration and magnitude did not present any significant variation between the two conditions, suggesting a similar PKC. This result might first be explained by a lesser net muscular activity along the whole trunk in unilateral conditions, as the load is only applied on one side. It can also be assumed that the inherently sagittal characteristics of the STS kinematics and kinetics (Shepherd and Gentile, 1994), attested in this study by the absence of a typical CP along the medial lateral axis, may limit its sensitivity to a left-right asymmetry of muscular tension. Finally, it can also be hypothesized that the CNS has integrated the asymmetry of postural muscles activity, which was set before the « Go » signal, in the programming of the task, and tuned the postural adjustments accordingly. All these hypotheses need further experiments to be tested and refined.

### Increased Muscular Tension in a Clinical Context May Require a Reorganization of the STS Motor Program

As increased active muscular tension is a very frequent symptom in various pathologies of the musculo-skeletal (muscular spasms in sports medicine or in degenerative diseases of the joints) and central nervous (hemiplegia, Parkinson’s disease) systems, the results from this study are worth being interpreted in a clinical perspective. It can first be assumed that a pathological increase of trunk muscular tension, when it exceeds a given level, may require an adaptation of the APAs pattern in order to keep the same level of performance in the STS task. It can also be considered that a limited asymmetry of trunk muscular tension may induce a negligible effect on the STS task performance. However, as increased tension was experimentally induced on healthy participants in this study, it is necessary to take into account the possible interaction with other symptoms in a pathological context, which may lead to more prominent effects. This might be the case in Parkinson’s disease or stroke, which are both associated with other deficits and have proved to impair STS task performance (Inkster and Eng, 2004; Boukadida et al., 2015). Therefore, treating pathological muscular tensions, by means of drugs or physiotherapy, might reduce the necessity for APAs reprogramming and favor STS performance in some pathologies.

### Limitation

There were some methodological limitations of the study which need to be acknowledged. First, we used a model of increased muscular tension based on voluntary contractions performed in healthy subjects, which intrinsically differ from pathological variations related to deficits of the nervous or musculo-skeletal systems. Second, the STS task was performed at maximum velocity in young adults, whereas slower movements are frequently observed in a pathological population, with possible variations of the focal and postural components of the task. Third, we only used participants with a normal BMI, whereas the STS task requires the generation of high propulsive forces that may lead to different strategies in subjects presenting high BMI. For these reasons, future experiments are needed to confirm our hypotheses in a more realistic context, including different pathologies and classes of participants.

## Conclusion

The findings of this study support the view that experimentally increased muscular tension along the trunk requires an adaptation of the APAs, which become longer, to maintain the same level of performance in the STS. This effect, which might be more prominent in a clinical context where it is bound be associated with other symptoms, may have some implications in treatment strategies intended to improve functional autonomy.

## Author Contributions

AH contributed with project creation, data analysis, and drafted the manuscript. CA-R contributed with project creation, data collection and data analysis. AH and CA-R discussed the results and revised the manuscript.

## Funding

This work was funded by University JF Champollion and Conseil Régional Occitanie, France.

## Conflict of Interest Statement

The authors declare that the research was conducted in the absence of any commercial or financial relationships that could be construed as a potential conflict of interest. The handling Editor currently co-hosts a Research Topic with one of the authors AH, and confirms the absence of any other collaborations. He states that the process met the standards of a fair and objective review.
